# Salt Eustress Induction in Red Amaranth (*Amaranthus gangeticus*) Augments Nutritional, Phenolic Acids and Antiradical Potential of Leaves

**DOI:** 10.3390/antiox11122434

**Published:** 2022-12-09

**Authors:** Umakanta Sarker, Sezai Ercisli

**Affiliations:** 1 Department of Genetics and Plant Breeding, Faculty of Agriculture, Bangabandhu Sheikh Mujibur Rahman Agricultural University, Gazipur 1706, Bangladesh; 2 Department of Horticulture, Faculty of Agriculture, Ataturk University, Erzurum 25240, Turkey

**Keywords:** *A. gangeticus*, protein and dietary fiber, minerals, phytochemicals, HPLC-UV DPPH, ABTS+, PA profiles, NaCl

## Abstract

Earlier researchers have highlighted the utilization of salt eustress for boosting the nutritional and phenolic acid (PA) profiles and antiradical potential (ARP) of vegetables, which eventually boost food values for nourishing human diets. Amaranth is a rapidly grown, diversely acclimated C_4_ leafy vegetable with climate resilience and salinity resistance. The application of salinity eustress in amaranth has a great scope to augment the nutritional and PA profiles and ARP. Therefore, the *A. gangeticus* genotype was evaluated in response to salt eustress for nutrients, PA profile, and ARP. Antioxidant potential and high-yielding genotype (LS1) were grown under four salt eustresses (control, 25 mM, 50 mM, 100 mM NaCl) in a randomized completely block design (RCBD) in four replicates. Salt stress remarkably augmented microelements, proximate, macro-elements, phytochemicals, PA profiles, and ARP of *A. gangeticus* leaves in this order: control < low sodium chloride stress (LSCS) < moderate sodium chloride stress (MSCS) < severe sodium chloride stress (SSCS). A large quantity of 16 PAs, including seven cinnamic acids (CAs) and nine benzoic acids (BAs) were detected in *A. gangeticus* genotypes. All the microelements, proximate, macro-elements, phytochemicals, PA profiles, and ARP of *A. gangeticus* under MSCS, and SSCS levels were much higher in comparison with the control. It can be utilized as preferential food for our daily diets as these antiradical compounds have strong antioxidants. Salt-treated *A. gangeticus* contributed to excellent quality in the end product in terms of microelements, proximate, macro-elements, phytochemicals, PA profiles, and ARP. *A. gangeticus* can be cultivated as an encouraging substitute crop in salt-affected areas of the world.

## 1. Introduction

Amaranth is a promising millennium vegetable with vast diversity [1,2,3,4,5,6,7]. It is an alternate source of nutrients because of its richness in vitamin C, minerals [8,9,10,11,12,13,14,15], vitamins [16,17,18,19,20], protein [21,22], dietary fiber [23,24,25], leaf pigments [26,27,28,29,30,31,32,33,34,35,36,37,38,39,40,41,42], phenolic compounds [43,44,45,46,47,48,49,50,51,52,53,54,55,56,57,58], and flavonoids [59,60,61,62,63,64,65,66,67,68,69,70,71,72,73] with strong antioxidants [74,75,76,77,78,79,80,81,82,83,84,85,86]. Amaranth has a noteworthy contribution as an antioxidant in food manufacturing owing to quenching reactive oxygen species (ROS) [87,88]. Wahid and Ghazanfar [89] reported that extreme salt enhanced the secondary plant metabolites, eventually accelerating plant protection apparatuses against ROS. Salinity enhances ROS production, which causes the oxidation of cellular components. ROS [90]. In plants, antioxidants (non-enzymatic), such as proteins, flavonoids, carbohydrates, carotenoids, and phenolic compounds, and enzymatic antioxidants are capable of ROS detoxification [90,91]. Hence, in human life, salt-tolerant plants could be considered a source of potent antioxidants. These compounds have extraordinary benefits to our food owing to quenching ROS and protecting against numerous diseases, such as cancer, cardiovascular diseases, atherosclerosis, cataracts, retinopathy, emphysema, arthritis, and neuron-damaging diseases [88].

Taste, flavor, and color determine the suitability of foods. Recently, consumers are very much interested in coloring food products. These products have much interest in the nutritional, safety, and beautification aspects of customers as foods. The utilization of natural pigments is considerably increasing day by day. The selected *A. gangeticus* genotype had sufficient betalains with bright red-violet color. *Amaranthus* leafy vegetable is an exclusive origin of betalains with significant quenching capacity of free radicals [92]. In low-acid foods, betalains are preferable to be utilized as a food colorant. These have greater stability than anthocyanins for pH [93], have preferential utility in the promotion of health, act as anti-inflammatory compounds, and diminish the risk of cancers of the skin and lungs and cardiovascular diseases. 

Amaranth is an extensively acclimated leafy vegetable due to diverse stresses, such as salinity [94,95,96] and drought [97], as well as having multiple uses. Salinity stress is a pioneer for the rapid augmentation of the quantity and quality of natural antioxidants through diverse factors, such as physiological, environmental, ecological, biological, biochemical, and evolutionary processes [98]. Very limited reports on the effect of salinity stress are available in terms of minerals, proximate, and bioactive compounds in different crops including leafy vegetables. Petropoulos et al. [99] reported the salinity-induced reduction of chlorophylls, fat, sugar, and carbohydrate and the augmentation of flavonoids, ascorbic acid (AsA), phenolics, proteins, and ARP in *Cichorium spinosum*. Different concentrations of sodium chloride enhanced the carotenoid content in buckwheat sprouts in comparison to the control [100]. Alam et al. [101] reported salt-induced amelioration of phenolics, ARP, and flavonoids in purslane. Ahmed et al. [102] recorded a salinity-induced increase in ARP and phenolics in barley. The influence of sodium chloride stress on the phytochemicals, nutrients, ARP, and PA profiles in *A. gangeticus* was studied for the first time. Based on our previous studies, the ARP genotype (accession LS1) along with high yield potential were selected. Therefore, the response of sodium chloride stress was assessed in *A. gangeticus* in terms of phytochemicals, nutrients, ARP, and PA profiles.

## 2. Materials and Methods

### 2.1. Experimental Site, Conditions, and Plant Materials

A high-yielding ARP genotype (accession LS1) was selected from among 120 genotypes from the Department of Genetics and Plant Breeding’s collection. The seeds were sown in four replicates following a block design with complete randomization (RCBD) in plastic pots at the Bangabandhu Sheikh Mujibur Rahman Agricultural University (24°23′ N, 90°08′ E, 8.4 m.s.l., AEZ-28 [103,104]. Pots were filled with sandy loam soil. P_2_O_5_:K_2_O was applied @ 48:60 kg ha^−1^ during the final land preparation. However, N was applied @ 46 kg ha^−1^ in two equally split doses during the final land preparation and 10 days after the sowing of the seeds. Four salt treatments, 100 (severe sodium chloride stress, SSCS), 50 (moderate sodium chloride stress, MSCS), and 25 (low sodium chloride stress, LSCS) mM NaCl, and a control (normal water) were used in the study. Pots were regularly irrigated with normal water for 10 days after sowing (DAS). At 11 DAS, salt treatments were imposed and sustained until the edible stage (30 DAS). Pots were irrigated once a day using salt water (100, 50, and 25 mM NaCl) and normal water. *A. gangeticus* leaves were harvested at 30 DAS.

### 2.2. Chemicals

Acetone, HClO_4_, HNO_3_, Sb, dithiothreitol (DTT), CsCl, AsA, 2, 2-dipyridyl, Trolox, PAs, HPLC grade acetonitrile, acetic acid, gallic acid (GAA), NaOH, rutin, DPPH, H_2_SO_4_, Folin-Ciocalteu reagent, MeOH, ABTS^+^, AlCl_3_.6H2O, Na_2_CO_3_, CH_3_CO_2_K, and K_2_S_2_O_8_. All chemicals were bought from Kanto Chemical Co. Inc. (Tokyo, Japan) and Merck (Germany).

### 2.3. Ash, Fiber, Moisture, Fat, Gross Energy, Carbohydrate, and Protein Estimation

The ash, fiber, moisture, fat, gross energy, and protein were estimated by the AOAC method [105,106,107]. The mini-Kjeldahl method was followed to measure nitrogen (N). Protein was calculated by multiplying N with 6.25. Protein, ash, fat, and moisture (%) were deducted from 100 to estimate carbohydrates.

### 2.4. Elements Estimation

The leaves were dried in an oven at 70 °C temperature for 24 h. Mineral elements were determined from the ground leaf by digesting with HNO_3_ and perchloric acid [105,108]. Exactly 0.5 g of the leaf samples were digested with 400 mL HNO_3_ (65%), 40 mL HClO_4_ (70%), and 10 mL H_2_SO_4_ (96%). The absorbance was read at 213.9 (Zn), 285.2 (Mg), 766.5 (K), 279.5 (Mn), 248.3 (Fe), 258.056 (S), 422.7 (Ca), 880 (P), 589 (Na), 430 (B), 313.3 (Mo), and 324.8 (Cu) nm wavelengths using an atomic absorption spectrophotometer (AAS with flame) (Hitachi, Japan). Macro- and micro-elements were expressed in mg g^−1^ and µg g^−1^ FW.

### 2.5. Beta-Carotene 

In a mortar and pestle, 500 mg leaves (fresh) were thoroughly mixed with 10 mL acetone (80%). The mixture was centrifuged at 10,000× *g* for 3–4 min for β-carotene determination [109]. After the separation of the filtrate in a flask, the final volume of 20 mL was maintained. The absorbance was taken at 510 and 480 nm by spectrophotometer (Tokyo, Japan). β-Carotene was expressed in fresh weight as mg 100 g^−1^.

### 2.6. Ascorbic Acid (AsA) Estimation 

Fresh leaves were used to determine AsA and DHA. The sample was pre-incubated by dithiothreitol (DTT), which reduced DHA to AsA. With the reduction of AsA, Fe^3+^ converted to Fe^2+^. Fe^2+^ complexes were formed by reacting Fe^2+^ and 2, 2-dipyridyl [109]. The absorbance of the complexes was taken at 525 nm by a spectrophotometer (Hitachi, Japan) to measure AsA in mg 100 g^−1^.

### 2.7. Samples Extraction and Determination of Total Polyphenols (TP), Total Flavonoids (TF), and Antiradical Potential (ARP)

Leaves were dried in a shady place to avoid direct sunshine. The extraction was performed from both the ground dried and fresh leaves (30 d) separately with a mortar and pestle. Total polyphenols (TP) were measured from fresh leaves, while total flavonoids (TF) content and ARP were determined from dried leaves. A 90% MeOH solution 10 mL was added with 0.25 g samples in a capped bottle tightly. The mixture was placed for 1 h in a shaker (Tokyo, Japan) at 60 °C. The final filtrate was stored for TP, TF, and ARP estimation. TF and TP were estimated by the AlCl_3_ colorimetric method and the Folin-Ciocalteu reagent, respectively [105,110]. The absorbance at 760 and 415 nm with a spectrophotometer (Hitachi, Japan). TP and TF were measured as GAA and rutin equivalent μg GAE g^−1^ of FW and μg RE g^−1^ DW using standard GAA and rutin curves. The Trolox equivalent antioxidant activity (TEAC) of ARP was estimated by the DPPH reduction and the ABTS^+^ assay [105,111]. ABTS^+^ and DPPH reduction percentage equivalent to the control was measured for estimating the ARP using the equation:

ARP (%) = (Ac − As/Ac) × 100

where Ac denotes the control absorbance (150 μL MeOH for ARP (ABTS) and 10 µL MeOH for ARP (DPPH) instead of leaf extract) and As is the absorbance of the samples. The results were calculated as μg Trolox equivalent g^−1^ DW.

### 2.8. Samples Extraction and Determination of Phenolic Acids (PAs) by HPLC

Fresh leaves (1 g) were extracted in MeOH (10 mL, 80%) containing CH₃COOH (1%). The thoroughly homogenized mixture was kept in a 50 mL tightly capped test tube and placed in a shaker (Scientific Industries Inc., New York, NY, USA) for 15 h at 400 rpm. It was filtered in a 0.45 µm filter (MA, New York, USA) and centrifuged for 15 min at 10,000× *g*. The filtrate was used to estimate PAs. All extractions were repeated 3 times. The method of Sarker and Oba [112] was followed to determine PAs using HPLC. Shimadzu HPLC (Kyoto, Japan) was furnished with a binary pump, degasser, and detector. A column (150 × 4.6 mm, 5 µm; Shinwa Chemical Industries, Ltd., Kyoto, Japan) was used for the separation of PAs. Solvent B and solvent A (acetonitrile and 6% (*v*/*v*) acetic acid in water, respectively) were pumped for 70 min at 1 mL min^−1^. HPLC system was run using a gradient program with 0–15% acetonitrile for 45 min, 15–30% for 15 min, 30–50% for 5 min, and 50–100% for 5 min; 35 °C temperature in the column was maintained with a 10 µL volume of injection. For monitoring Pas continuously, the detector was set at 254 and 280 nm. The retention time and UV–vis spectra with their respective standards were compared for the identification of the compound. Pas was estimated as µg g^−1^ FW.

Each PA was quantified using the corresponding standards of calibration curves. A total of 16 PAs were dissolved in MeOH (80%) 100 mg mL^−1^ as stock solutions. Individual PAs were quantified using corresponding standard curves (10, 20, 40, 60, 80, and 100 µg mL^−1^) with external standards. Retention times, co-chromatography of samples spiked with commercially available standards, and UV spectral characteristics were utilized for identification and matching the PA.

### 2.9. Statistical Analysis

All the sample data of a trait were averaged for each treatment to obtain a replication mean [113,114,115]. The mean data of various traits were statistically and biometrically analyzed [116,117,118]. Data analysis and ANOVA were performed using Statistix software version 8.0, Tallahassee FL 32312, USA [119,120,121]. The means were compared at a 1% level of probability using Duncan’s Multiple Range Test (DMRT). The results were reported as the mean ± SD of four separate replicates [122,123,124].

## 3. Results and Discussion

### 3.1. The Response of Proximate Compositions to Sodium Chloride Stress

**Figure 1:**
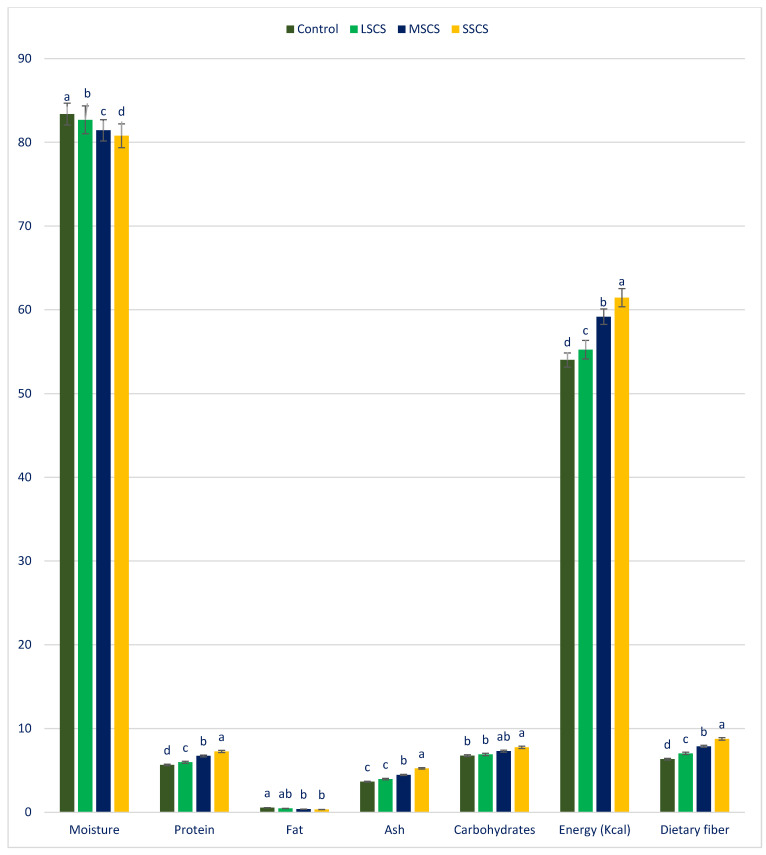
The response of ash, fiber, moisture, fat, gross energy, carbohydrate, and protein (g 100 g^−1^) to control, LSCS, MSCS, and SSCS in *A. gangeticus* accession; (n = 6), different letters in columns are varied significantly by Duncan Multiple Range Test (DMRT) (*p* < 0.01).

Figure 1 represents the nutritional compositions of *A. gangeticus* under different salinity stresses. *A. gangeticus* leaves had a high moisture content like most leafy vegetables. Nevertheless, our study revealed that *A. gangeticus* leaves have copious ash, carbohydrates, dietary fiber, moisture, and protein. The constituents of these components were several times greater than *C. spinosum* [99]. The maximum moisture and fat were exhibited under the control treatment, whereas the minimum moisture and fat were observed under SSCS. Petropoulos et al. [99] reported a similar reduction in fat with the increase in salinity stress in *C. spinosum.* Moisture and fat were significantly reduced in the order: (control > LSCS > MSCS > SSCS) and (control > LSCS > MSCS = SSCS), respectively. Higher leaf dry matter obtained from leaves ensure lower moisture content. Hence, salt-stressed *A. gangeticus* leaves confirmed greater dry matter in comparison to the control. The maximum dietary fiber, ash, carbohydrates, energy, and protein were recorded at SSCS, while the minimum dietary fiber, ash, carbohydrates, energy, and protein were noticed under the control. Similarly, Petropoulos et al. [99] reported higher ash and protein at the maximum and 8.0 and 6.0 dS m^−1^, than the control and minimum salinity in *C. spinosum*. Energy, protein, and dietary fiber contents were sharply augmented in the following order: control < LSCS < MSCS < SSCS, whereas ash and carbohydrates contents were statistically similar in the control and LSS levels and progressively augmented from MSCS to SSCS levels.

**Figure 2:**
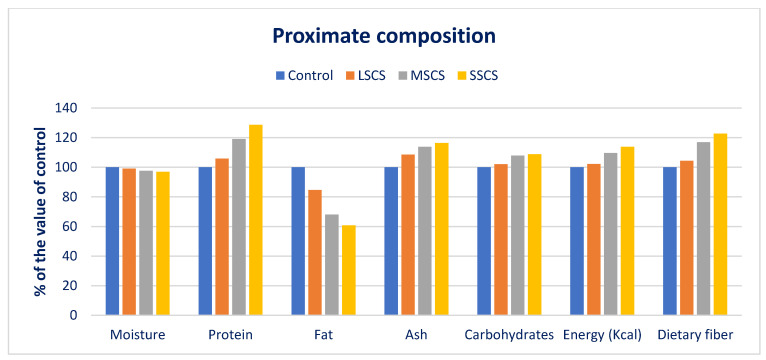
Changes of ash, fiber, moisture, fat, gross energy, carbohydrate, and protein over control in *A. gangeticus* accession.

In LSCS, MSCS, and SSCS, dietary fiber, energy, carbohydrates, ash, and protein were increased by 17%, 2%, 2%, 9%, and 4%; 6%, 10%, 8%, 14%, and 19%; and 23%, 14%, 9%, 16%, and 29%, respectively, in comparison with the control condition (Figure 2). 

Dietary fiber has significantly acted in the remedy of constipation, increased digestibility, and palatability. Vegetarians and deprived rural communities in underdeveloped countries mostly trust *A. gangeticus* for protein. Since the low amounts consumed in a daily diet, the increments of energy content in the order of control < LSCS < MSCS < SSCS had no substantial influence on the energy balance in humans. The findings of *A. gangeticus* conformed with the outcomes of AT [91] and leaves of *Ipomoea batata* [125], respectively. They specified that it influences cell function, the fat covering the body’s organs, and continues the temperature of the body. The fats of vegetables are prime sources of crucial fatty acids, such as Ω-6 and Ω-3. Fats perform a noteworthy contribution to the absorption, digestion, and transportation of vitamins A, E, K, and D.

### 3.2. Sodium Chloride Impact on Minerals (Macroelements and Microelements) Composition

**Figure 3:**
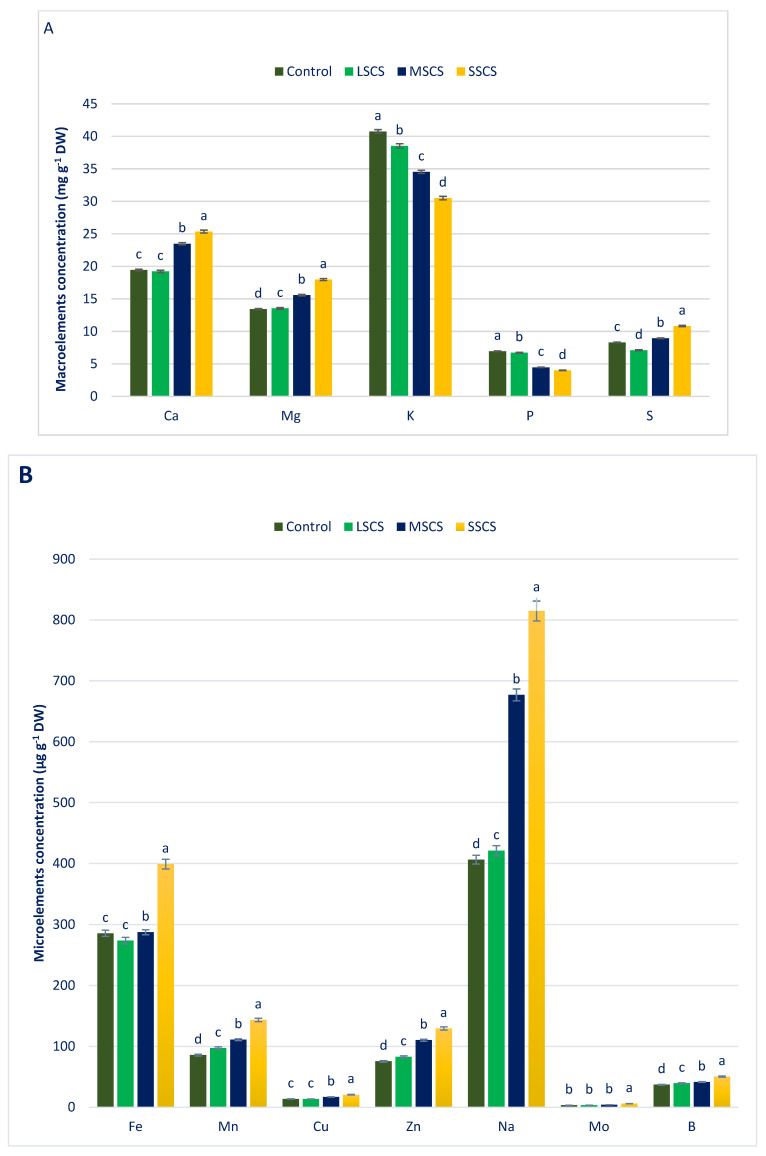
Response of minerals concentration (**A**) macroelements and (**B**) microelements under control, LSCS, MSCS, and SSCS in *A. gangeticus* accession; (n = 6), different letters in columns are varied significantly by DMRT (*p* < 0.01).

*A. gangeticus* has abundant minerals (macroelements and microelements) (Figure 3). High levels of minerals were observed and corroborated with *A. tricolor* under normal cultivation practice in an open field [126]. *A. gangeticus* had higher Fe and Zn than *Manihot esculenta* leaves [127] and *Lathyrus japonicus* [128]. Jimenez-Aguiar and Grusak [129] also found abundant Zn, Cu, Mn, and Fe in different *A. spp.* They also found higher iron and copper compared with kale and higher Zn compared with leaf cabbage, *Spinacia oleracea*, and *Solanum nigrum*. The maximum Zn, Ca, Mo, Mg, Na, S, Cu, B, Mn, and Fe was noticed under the SSCS level, while the minimum levels Zn, Ca, Mo, Mg, Na, Cu, B, Mn, and Fe were reported under control conditions, and the lowest sulfur content was observed under the LSCS level. Zn, Ca, Mo, Mg, Na, Cu, B, and Mn were progressively augmented in the order control < LSCS < MSCS < SSCS. In contrast, potassium and phosphorus contents were drastically reduced in the order control > LSCS > MSCS > SSCS. 

**Figure 4:**
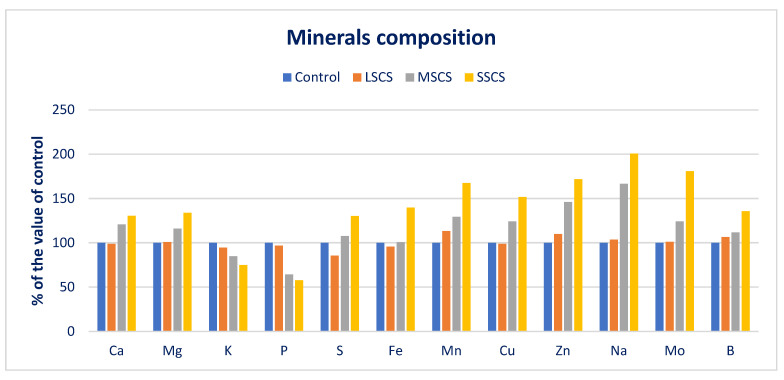
Response of minerals (macroelements and microelements) over control in *A. gangeticus* accession.

In LSCS, MSCS, and SSCS, Zn, Ca, Cu, Mo, Mg, Mn, B, and Na were augmented by −1%, 0.8%, 13%, −1%, 10%, 4%, 1%, and 6%; 21%, 16%, 29%, 24%, 46%, 67%, 24%, and 12%; and 30%, 34%, 67%, 52%, 72%, 100%, 81%, and 36%, respectively, in comparison with the control condition (Figure 4). In LSCS, MSCS, and SSCS, potassium and phosphorus content declined to 5%, 14%, 25%, and 3%, 36%, 42%, respectively, in comparison with the control condition (Figure 4). 

Most of the minerals increased under different salt levels compared with control conditions, which were corroborated with minerals of *C. spinosum* under salinity stress [99]. Petropoulos et al. [99] reported sharp augmentation in calcium, magnesium, iron, manganese, zinc, and sodium content and a reduction in potassium content in *C. spinosum*. They stated that the application of fertilizer and treatments of salinity could be the reason for the amelioration of sodium content and suggested that the species utilized accumulated sodium to cope with the adverse effects of salinity. Iron content was statistically similar to the value of the control and LSCS levels, while iron content was progressively augmented under MSCS and SSCS levels by 12% and 62%, respectively. The lowest sulfur content was obtained from the LSCS levels, which differed significantly from the control condition. The sulfur content was gradually augmented under MSCS and SSCS levels by 20% and 51%, respectively (Figure 4).

### 3.3. Impact of Salinity on Phytochemicals and ARP

**Figure 5:**
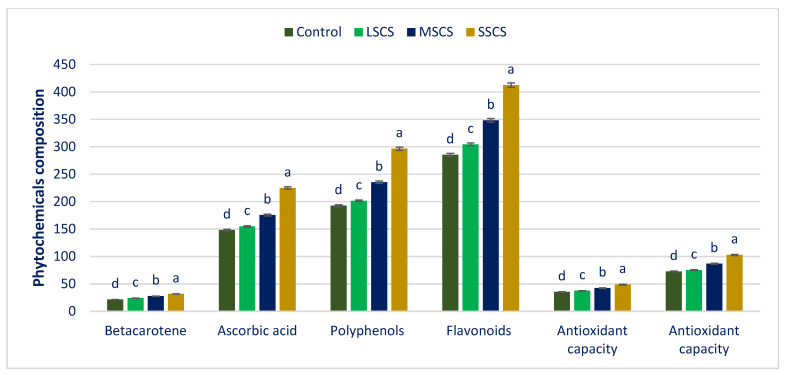
Effect of salinity treatments (control, LSCS, MSCS, and SSCS) on phytochemicals composition in *A. gangeticus* accession. Flavonoids (µg RE g^−1^ DW), AsA and beta-carotene (mg 100 g^−1^ FW), ARP (DPPH and ABTS^+^) (µg TEAC g^−1^ DW), and polyphenols (µg GAE g^−1^ FW), (n = 6); different letters in columns are varied significantly by DMRT (*p* < 0.01).

Polyphenols, beta-carotene, AsA, flavonoids, and ARP varied significantly under different sodium chloride stresses (Figure 5). Sodium chloride stress progressively augmented polyphenols, beta-carotene, AsA, flavonoids, and ARP in the following order: control < LSCS < MSCS < SSCS. 

**Figure 6:**
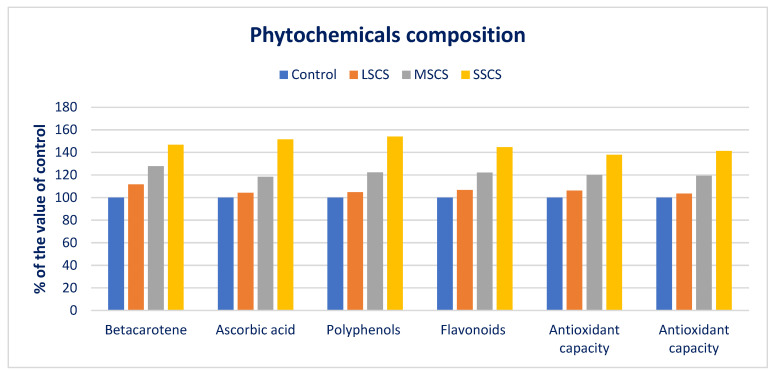
Comparison of phytochemicals over control in *A. gangeticus* accession.

Beta-carotene, AsA, polyphenols, flavonoids, and ARP (DPPH and ABTS^+^) under LSCS, MSCS, and SSCS were predominately augmented by 12%, 4%, 5%, 7%, 6%, and 3%; 28%, 18%, 22%, 22%, 20%, and 19%; and 47%, 52%, 54%, 45%, 38%, and 41% than control, respectively (Figure 6). 

The maximum polyphenols, beta-carotene, flavonoids, AsA, and ARP (DPPH and ABTS^+^) were recorded under SSCS. Conversely, the lowest polyphenols, beta-carotene, flavonoids, AsA, and ARP (DPPH and ABTS^+^) were confirmed under the control. Petropoulos et al. [99] reported the salinity-induced augmentation of flavonoids, ARP, AsA, and phenolics in *C spinosum*. Different concentrations of sodium chloride enhanced the carotenoid content in buckwheat sprouts in comparison with the control (Lim et al. [100]. Alam et al. [101] reported salt-induced amelioration of phenolics, ARP, and flavonoids in purslane. In barley, a similar salinity-induced increase of ARP and phenolics were stated.

### 3.4. Response of Salinity on PA Profiles

The HPLC-identified PA values of *A. gangeticus* (accession LS11) under four sodium chloride stress were compared with PAs using the respective peaks of the compounds (Table 1). Sixteen PAs, including seven CAs and nine Bas, were confirmed in *A. gangeticus*. Three BAs [protocatechuic acid (PCA), β-resorcylic acid (β-RA), and gentisic acid (GA)] were identified as new compounds for the first time in *Amaranthus* leaves. 

**Figure 7:**
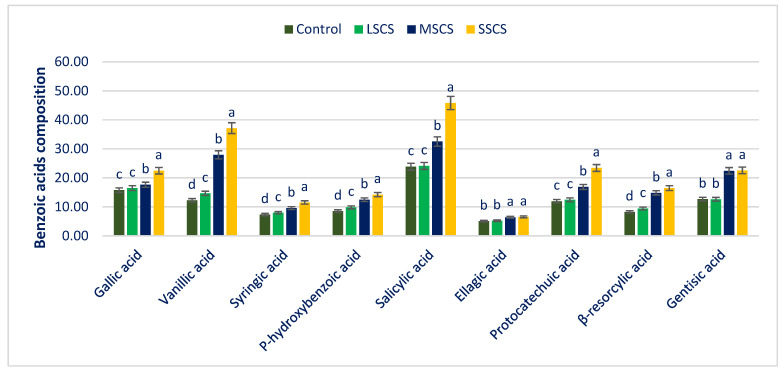
Impact of BAs concentrations (µg g^−1^ FW) under control, LSCS, MSCS, and SSCS in *A. gangeticus* accession; (n = 6), different letters in columns are varied significantly by DMRT (*p* < 0.01).

BAs were the amplest among the two categories of acids, thereafter CAs in *A. gangeticus* (Figure 7 and Figure 9). Salicylic acid (SA) was the most copious PAs across BAs thereafter GAA, GA, PCA, vanillic acid (VA), *p*-hydroxybenzoic acid (*p*-HBA), β-RA, and syringic acid (SYA) (Figure 7). BA contents in the *A. gangeticus* genotype under control conditions were superior to the BA content of *A. tricolor* [130]. Chlorogenic acid (CHA) was the most noticeable compound across CAs thereafter ferulic acid (FA), sinapic acid (SIA), *m*-coumaric acid (*m*-COA), *trans*-cinnamic acid (*Trans*-CA), and caffeic acid (CFA) (Figure 7). *A. gangeticus* had abundant CAs under control conditions. Seven CAs obtained were confirmed superior to CAs of *A. tricolor* [130]. Phenylalanine is the most extensively distributed PA in plant tissues, which are finally synthesized into CAs [131]. Identified Benzoic acids (BAs) have important biological activities. For instance, gallic acid and its ester derivatives ARE flavoring agents and preservatives in the food industry. There are diverse scientific reports on the biological and pharmacological activities of these phytochemicals, with emphasis on antioxidant, antimicrobial, anti-inflammatory, anticancer, cardioprotective, gastroprotective, and neuroprotective effects [132]. Vanillic acid exerts diverse bioactivity against cancer, diabetes, obesity, neurodegenerative, cardiovascular, and hepatic diseases by inhibiting the associated molecular pathways. Its derivatives also possess the therapeutic potential to treat autoimmune diseases, as well as fungal and bacterial infections [133]. Syringic acid shows a wide range of therapeutic applications in the prevention of diabetes, CVDs, cancer, and cerebral ischemia, as well as antioxidant, antimicrobial, anti-inflammatory, antiendotoxic, neurologic, and hepatoprotective activities [134]. High salicylate in diets has proven health benefits, such as lower risks of cancer, heart disease, and diabetes. Ellagic acid has been reported to have antimutagenic on bacteria and in mammalian systems as well. It has also shown strong antioxidant, anti-inflammatory, and anticarcinogenic activities, as well as a better preservative effect against oxidative stress when compared with vitamin E [135]. PCA is a major metabolite of anthocyanin. The pharmacological actions of PCA have been shown to include strong in vitro and in vivo antioxidant activity. In in vivo experiments using rats and mice, PCA has been shown to exert anti-inflammatory as well as antihyperglycemic and antiapoptotic activities [136]. β-resorcylic acid has antimicrobial activity [137]. Finally, gentisic acid possesses fibro growth factor inhibition, antimicrobial, antioxidant, anti-inflammatory, hepatoprotective, and neuroprotective activities [138].

**Figure 8:**
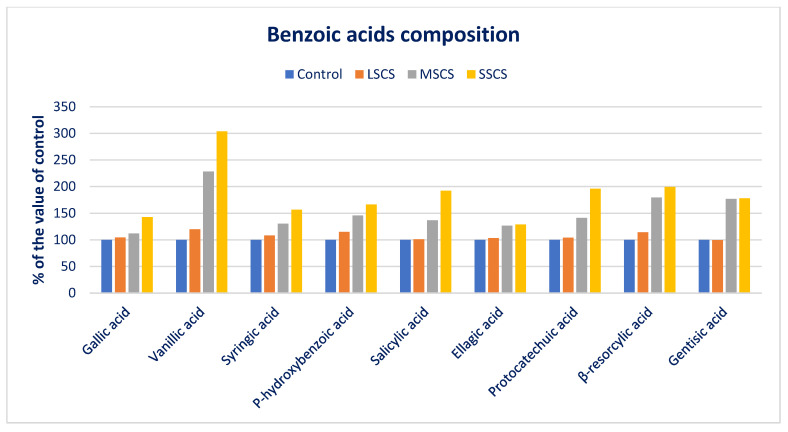
Comparison of BAs composition over control in *A. gangeticus* accession.

Sodium chloride stress predominately augmented all the BA compositions. At SSCS, all the BAs displayed the maximum contents, while the minimum BA contents were obtained from the control treatment. From control to SSCS, VA, β-RA, *p*-HBA, and SYA ranged from 12.24 to 37.15, 8.26 to 16.48, 8.55 to 14.23, and 7.36 to 11.52 µg g^−1^ FW, respectively (Figure 7). VA, β-RA, *p*-HBA, and SYA progressively augmented in the order: Control < LSCS < MSCS < SSCS (Figure 7). VA, β-RA, *p*-HBA, and SYA under LSCS, MSCS, and SSCS were predominately augmented by 20%, 28%, 204%; 14%, 79%, 100%; 15%, 46%, and 66%; and 8%, 30%, and 57% than control, respectively (Figure 8). 

SA, GAA, and PCA contents had no statistical variations at the control and LSCS level; however, three acids were augmented remarkably from LSCS to SSCS with a range from 23.83 to 45.82, 15.76 to 22.46, and 11.95 to 23.42 µg g^−1^ FW, respectively (Figure 7). In MSCS and SSCS, SA, GAA, and PCA contents were augmented by (37% and 92%), (12% and 43%), and (41% and 96%), respectively (Figure 8). GA and ellagic acid (EA) ranged from 12.68 to 22.58 and 5.08 to 6.55 µg g^−1^ FW. GA and EA had no statistical variations between control and LSCS levels and between MSCS and SSCS levels; however, the contents of these acids were augmented remarkably from control condition or LSCS to MSCS or SSCS level (26% and 77%) (Figure 7 and Figure 8).

**Figure 9:**
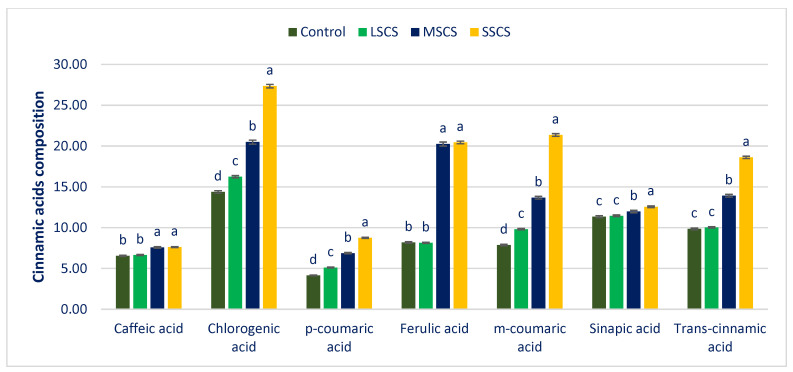
Response of CAs composition (µg g^−1^ FW) under control, LSCS, MSCS, and SSCS in *A. gangeticus* accession; (n = 6), different letters in columns are varied significantly by DMRT (*p* < 0.01).

All the CA contents were sharply augmented under sodium chloride levels. All the CAs showed the highest contents under the SSCS level, whereas the control treatment exhibited the lowest CA contents. From control to SSCS, CHA, *m*-COA, and *p*-coumaric acid (*p*-COA) ranged from 14.38 to 27.35, 7.87 to 21.36, and 4.16 to 8.75 µg g^−1^ FW, respectively, (Figure 9). Identified Cinnamic acids (CAs) have important biological activities. For instance, Caffeic acid (CA) and its derivatives have antioxidant, anti-inflammatory, and anticarcinogenic activity [139]. Chlorogenic acid was effective in preventing weight gain, inhibiting the development of liver steatosis, and blocking insulin resistance induced by a high-fat diet [140]. *p*-coumaric acid decreases low-density lipoprotein (LDL) peroxidation, shows antioxidant and antimicrobial activities, and plays an important role in human health [141]. Ferulic acid has low toxicity and possesses many physiological functions (anti-inflammatory, antioxidant, antimicrobial activity, anticancer, and antidiabetic effects). It has been widely used in the pharmaceutical, food, and cosmetics industries [142]. Sinapic acid shows antioxidant, antimicrobial, anti-inflammatory, anticancer, and anti-anxiety activity [143]. Cinnamic acids have been identified as interesting compounds with antioxidant, anti-inflammatory, and cytotoxic properties [144].

**Figure 10:**
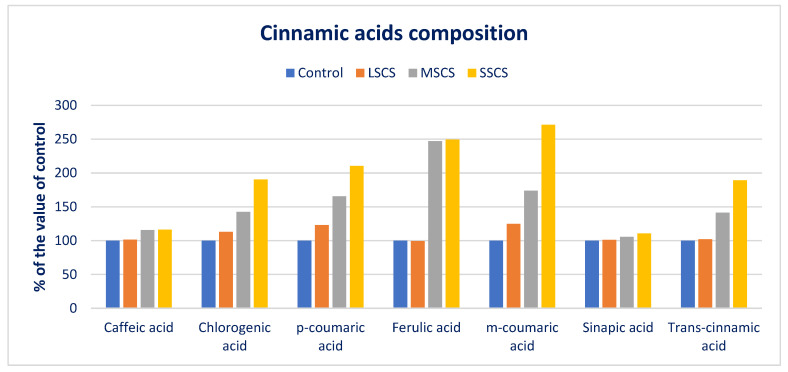
Comparison of CAs over control in *A. gangeticus* accession.

CHA, *m*-COA acid, and *p*-COA were progressively augmented in the order of control < LSCS < MSCS < SSCS (Figure 9). In LSCS, MSCS, and SSCS, CHA, *m*-COA acid, and *p*-COA were predominately augmented by 13%, 42%, 90%; 25%, 74%, 171%; and 23%, 65%, 110% compared with the control condition, respectively (Figure 10). *Trans*-CA and SIA contents at control condition were statistically similar to the LSCS level; however, these two acids’ contents were remarkably augmented from LSCS to SSCS with a range from 9.85 to 18.62 and 11.35 to 12.56 µg g^−1^ FW, respectively (Figure 9). In MSCS and SSCS, *Trans*-CA and SIA contents were augmented by 41% and 89%; and 6% and 11%, respectively (Figure 10). FA and CFA ranged from 8.20 to 20.45 and 6.56 to 7.62 µg g^−1^ FW, respectively. FA and CFA contents at the control condition were statistically similar to the LSCS level, and at the MSCS level were statistically similar to the SSCS level. However, the contents of these acids were remarkably augmented from the control condition or LSCS to MSCS or SSCS level (47% and 16%) (Figure 9 and Figure 10).

**Figure 11:**
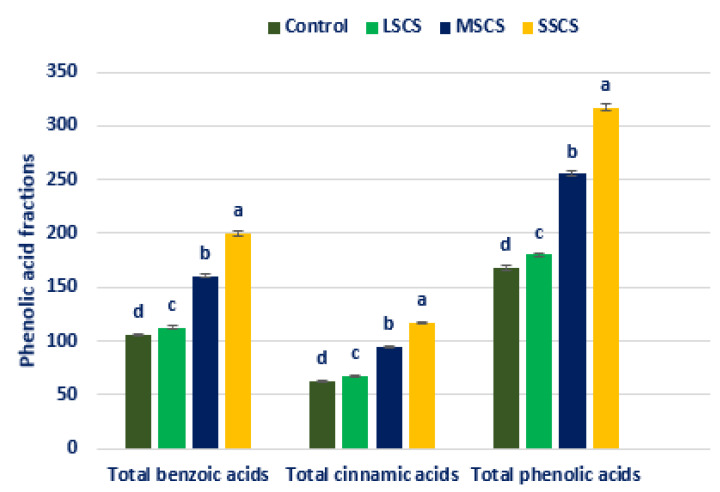
Increase of PA fractions (µg g^−1^ FW) (total BAs, total CAs, and total PAs) under control, LSCS, MSCS, and SSCS in *A. gangeticus* accession; (n = 6); different letters in columns are varied significantly by DMRT (*p* < 0.01).

All the PA fractions were sharply and remarkably augmented under sodium chloride stress. All the PA fractions exhibited the highest contents under SSCS level, whereas the control treatment had the lowest PA fractions. From control to SSCS, total BAs, total CAs, and total PAs ranged from 105.71 to 200.21, 62.37 to 116.71, and 168.08 to 316.92, µg g^−1^ FW, respectively (Figure 11). 

**Figure 12:**
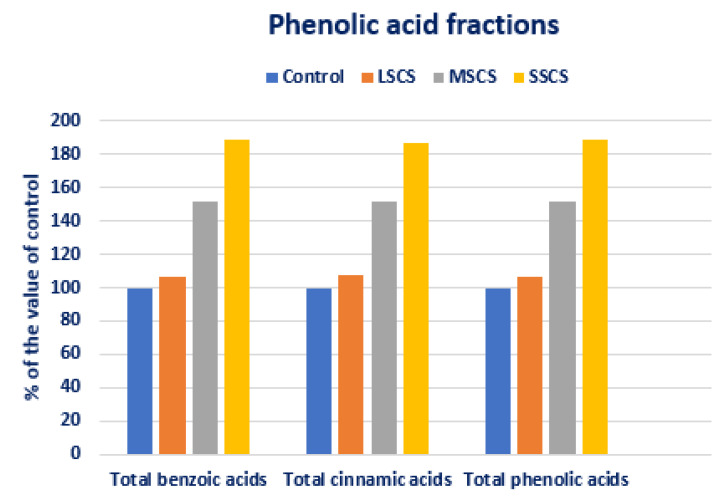
Comparison of phenolics acid fractions (total BA, total CAs, and total PAs) over control in *A. gangeticus* accession.

Total BAs, total CAs, and total PAs were progressively augmented in the order control < LSCS < MSCS < SSCS (Figure 11). In LSCS, MSCS, and SSCS, total BAs, total CAs, and total PAs were predominately augmented by 7%, 52%, and 89%), (8%, 52%, and 87%), and (7%, 52%, and 89%), compared with control condition, respectively (Figure 12). 

Petropoulos et al. [99] reported the salinity-induced augmentation of PAs in *C. spinosum*. Klados and Tzortzakis [145] showed a progressive increment of total PAs under increased sodium chloride stress in *C. spinosum*. Alam et al. [101] reported salt-induced amelioration of phenolics in purslane. Ahmed et al. [103] reported a salinity-induced increment of PA profiles in barley. In contrast, Neffati et al. [146] stated the reduction of PA profiles with an increment of sodium chloride concentrations in coriander.

The cost is very low to maintain salt stress by adding sodium chloride to the plants. Furthermore, we suggested cultivating in salt-prone areas where there are no salt susceptible crops grown successfully. So, those areas will be efficiently utilized for amaranth leafy vegetable cultivation to meet the demand for the leafy vegetable of that locality, as leafy vegetables are too susceptible to salinity stress as amaranth is a salinity-tolerant leafy vegetable with up to 200 mM salt concentration. It can produce enough biomass and perform optimal photosynthesis at 100 mM saline stress. Amaranth is highly tolerant to salinity. It can tolerate 200 mM NaCl [147]. As amaranth is salt tolerant, it increases all enzymatic and non-enzymatic antioxidants, and metabolites, to detoxify ROS and cope with salt stress.

## 4. Conclusions

Sodium chloride stress remarkably augmented the energy, ash, carbohydrates, protein, calcium, dietary fiber, magnesium, S, Fe, Mo, Mn, Na, Cu, B, Zn, and ARP of *A. gangeticus* leaves. All the nutrients, phytochemicals, PA profiles, and ARP of *A. gangeticus* leaves under MSCS and SSCS levels were superior to the control. It can be utilized as a valued product for human consumption and health benefits. Salt-treated *A. gangeticus* leaves had abundant nutrients, phytochemicals, PA profiles, and ARP. Phytochemicals, PA profiles, and ARP scavenge ROS that would be advantageous for human health benefits as these bioactive compounds have potent antioxidants. Furthermore, sodium chloride-stressed *A. gangeticus* contributed with excellent quality in the end users for nutrients, phytochemicals, PA profiles, and ARP. It can be cultivated as a promising substitute crop in sodium chloride-affected areas of the world.

## Figures and Tables

**Table 1:** Wavelengths of maximum absorption in the visible region (λ_max_), mass spectral data, retention time (Rt), and tentative identification of PAs in *A. gangeticus*.

Peak No	Rt (min)	λ_max_ (nm)	Molecular Ion [M-H]^−^ (*m*/*z*)	MS^2^ (*m*/*z*)	Identity of Tentative Phenolic Acids
1	9.1	254	169.1142	169.1563	3,4,5 Trihydroxybenzoic acid
2	30.6	254	167.1214	167.1564	4-Hydroxy-3-methoxybenzoic acid
3	34.8	254	197.1132	197.1104	3,5-Dimethoxy-4-hydroxybenzoic acid
4	31.5	254	137.0213	137.1574	4-Hydroxybenzoic acid
5	48.2	254	137.2113	137.1582	2-Hydroxybenzoic acid
6	52.5	254	301.0423	301.0643	2,3,7,8-Tetrahydroxy-chromeno [5,4,3-cde] chromene-5,10-dione
7	2.2	280	154.1212	154.1157	3,4-Dihydroxybenzoic acid
8	4.0	280	154.1212	154.0156	2,4-Dihydroxybenzoic acid
9	3.7	280	154.1212	154.1157	2,5- Dihydroxybenzoic acid
10	32.0	280	179.0821	179.0687	3,4-Dihydroxy-trans-cinnamate
11	31.1	280	353.1253	353.1542	3-(3,4-Dihydroxy cinnamoyl) quinic acid
12	42.0	280	163.0658	163.1241	4-Hydroxy cinnamic acid
13	47.9	280	193.1726	193.1649	3-Methoxy-4-hydroxy cinnamic acid
14	49.6	280	163.2547	163.2872	3-Hydroxy cinnamic acid
15	49.0	280	223.1568	223.1748	4-Hydroxy-3,5-dimethoxy cinnamic acid
16	67.3	280	147.1142	147.1103	3-Phenyl acrylic acid

## Data Availability

The data that are recorded in the current study are available in all of the tables and figures of the manuscript.
